# Impact of intraoperative parathyroid identification and accidental removal on post-thyroidectomy hypocalcemia: a single-center 15-year experience

**DOI:** 10.1186/s12893-026-03959-0

**Published:** 2026-06-18

**Authors:** A. Brugués, S. Eschlböck, E. Siegenthaler, S. Taha-Mehlitz, R. Sortino, T. Delko, R. Droeser, A. Posabella

**Affiliations:** 1 University Center of Gastrointestinal and Liver Diseases-Clarunis, University of Basel, Basel, Switzerland; 2 Faculty of Medicine, University of Basel, Basel, Switzerland; 3 Department of Surgery, Klinik St. Anna, Luzern, Switzerland

**Keywords:** Parathyroid, Intraoperative identification, Hypocalcemia, Postoperative complication

## Abstract

**Introduction:**

Post-thyroidectomy hypocalcemia remains a common and clinically significant complication. Despite advances in surgical technique and perioperative care, rates of transient and persistent hypocalcemia remain substantial, with reported transient rates up to 50% and persistent rates up to 4%. This study evaluates the impact of intraoperative parathyroid gland identification and accidental removal on postoperative hypocalcemia in a Swiss hospital.

**Methods:**

We conducted a retrospective analysis of 615 adult patients undergoing thyroid surgery at the University Hospital of Basel between 2007 and 2022. 322 patients (52.3%) who received total thyroidectomy were selected for statistical analysis. Data on demographics, intraoperative nerve monitoring, number of parathyroid glands visually identified, histological confirmation of gland removal and use of autotransplantation were collected. Postoperative hypocalcemia was categorized as temporary (< 6 months) or persistent (> 6 months). Statistical analyses included chi-squared tests, Wilcoxon rank-sum tests, and logistic regression to assess associations between gland identification/removal and hypocalcemia, with significance set at *p* < 0.05.

**Results:**

Among 288 patients who underwent total thyroidectomy with intraoperative identification of parathyroid glands, 43% (*n* = 124) developed temporary hypocalcemia and 0.9% (*n* = 3) experienced persistent hypocalcemia. Surgeons identified a median of 2.54 parathyroid glands intraoperatively, with no significant association emerging between the number of glands identified and temporary hypocalcemia (*p* = 0.594). Histological evidence of inadvertent gland removal occurred in 18.6% of cases and correlated with a non-significantly higher rate of temporary hypocalcemia (47.3% vs. 40.5%, *p* = 0.402). Parathyroid autotransplantation had a significantly higher rate of postoperative hypocalcemia compared with those without autotransplantation (61.0% vs. 38.6%; OR 2.48, 95% CI 1.26–4.87; *p* = 0.007).

**Conclusion:**

Our findings suggest that intraoperative visual identification of parathyroid glands alone may not be sufficient to reliably predict postoperative hypocalcemia, highlighting the multifactorial nature of postoperative calcium disturbances. Accidental gland removal increases temporary hypocalcemia but lacks statistical significance. Autotransplantation, while useful for preserving long-term function, may initially exacerbate transient hypocalcemia. Meticulous surgical techniques and preservation of gland vascularity remain paramount to minimize hypocalcemia risk. Future prospective studies should evaluate the role of novel intraoperative identification technologies in reducing these complications.

## Introduction

Thyroidectomy remains a key intervention in the treatment of both malignant and benign thyroid pathologies. Advances in surgical techniques and perioperative management have significantly improved patient outcomes, but complications persist. Postoperative hypocalcemia is still one of the most frequent and clinically significant issues. Despite its apparent predictability, the condition remains a challenge for surgeons and endocrinologists [1, 2]. Hypocalcemia after thyroidectomy is primarily driven by the disruption of parathyroid gland function. During surgery, these glands may be inadvertently removed, devascularized, or injured. Traditional methods for identifying parathyroid glands during surgery rely on the surgeon’s experience and extensive knowledge of neck anatomy [3]. Anatomical landmarks play a crucial role [4]. The extent of hypocalcemia varies, with transient forms typically resolving within a few weeks to months and persistent hypocalcemia persisting beyond six months, often requiring lifelong calcium and vitamin D supplementation [5]. Reported rates of transient hypocalcemia range widely, affecting between 20% and 50% of thyroidectomy patients, while persistent hypocalcemia is observed in 1–4% of cases [6]. Understanding the risk factors for hypocalcemia is critical for prevention and management. Preoperative vitamin D deficiency, older age, female sex, and underlying thyroid pathology, including Graves’ disease and malignancy, are associated with an increased likelihood of hypocalcemia [7, 8]. The preservation of parathyroid glands is critical, with studies showing that identifying and preserving at least one well-vascularized gland significantly reduces the risk of persistent hypoparathyroidism [9]. Advancements in surgical and diagnostic techniques such as parathyroid autotransplantation offer promising solutions for reducing the incidence of long-term postoperative hypocalcemia. Another valuable, relatively new tool is near-infrared autofluorescence (NIRAF), which is changing the paradigm in which thyroid surgery gets performed [10]. Its integration into thyroid surgery has been explored globally as studies worldwide have demonstrated its efficacy in enhancing parathyroid identification [11, 12]. This technique is however limited by factors such as variability in fluorescence intensity, interference from background tissues, shallow penetration depth, operator dependency, and limited universal availability, which collectively impact its consistency and utility in clinical practice [13]. This highlights the need to perform correct intraoperative parathyroid gland identification using traditional methods on a routine basis.

The aim of this study was to evaluate the association between intraoperative parathyroid gland identification, accidental parathyroid gland removal and parathyroid autotransplantation with the incidence of postoperative hypocalcemia following total thyroidectomy in a large retrospective cohort from a single center.

## Methods

This retrospective cohort study was conducted at the University Hospital of Basel and involved the analysis of health-related data from patients who underwent thyroid surgery between 2007 and 2022. Information on patient demographics, preoperative evaluations, surgical procedures, and postoperative outcomes was extracted and analyzed. The extent of surgery (total thyroidectomy, hemithyroidectomy, subtotal thyroidectomy and isthmectomy), use of intraoperative nerve monitoring, parathyroid gland identification and autotransplantation were recorded. An assessment of intra- and postoperative complications was performed. Intraoperative nerve injury was considered to be a loss of neuromonitoring signal, with or without visible damage to the recurrent laryngeal nerve during surgery, persisting for less than 6 months (temporary) or longer than 6 months (persistent). Postoperative bleeding was defined as hemorrhage requiring surgical revision. Other intraoperative complications included rare events such as esophageal injury or tracheal injury. Postoperative hypocalcemia was defined as corrected serum calcium below the institutional lower reference limit (< 2.10 mmol/L). According to biochemical calcium levels measured after the procedure hypocalcemia was classified as transient hypocalcemia (hypocalcemia occurring after surgery and resolving within 6 months) and persistent hypocalcemia (hypocalcemia persisting for more than 6 months after surgery, requiring ongoing calcium and/or vitamin D supplementation).

The study included adult patients (≥ 18 years old) who underwent thyroid surgery for thyroid-related conditions. Eligible thyroid conditions included autoimmune disorders, malignancies, chemically induced disorders, and thyroid dysfunction due to other causes. Patients who underwent surgery for parathyroid-related conditions and patients who formally refused consent for the use of their data were excluded from this study.

The main analysis of this study consisted of evaluating the relationship between intraoperative parathyroid gland identification, autotransplantation, histological confirmation and postoperative hypocalcemia in patients who underwent total thyroidectomy. While the entire cohort of thyroid surgical procedures was included for descriptive purposes, the main analysis on impact of intraoperative parathyroid gland identification, autotransplantation and accidental removal on postoperative hypocalcemia was restricted to patients undergoing total thyroidectomy. The number of parathyroid glands identified intraoperatively was recorded for each patient, ranging from 0 to 4 glands. The presence of parathyroid glands was determined by the operating surgeon using conventional visual identification techniques. The frequency of intraoperative parathyroid glands identification was categorized into five groups (0, 1, 2, 3, and 4 parathyroid glands identified), and statistical distributions were examined. Postoperative histological examination of the specimen was performed to determine whether parathyroid glands had been inadvertently removed. The presence of parathyroid gland in histological samples was recorded as 0 (no parathyroid gland found), 1 (one parathyroid gland identified), or 2 (two parathyroid glands identified). The incidence of postoperative hypocalcemia was compared with both intraoperative parathyroid gland identification and histological findings. Parathyroid autotransplantation was performed when the viability of a gland was considered compromised during surgery based on the quality of the gland itself. The gland was then minced and implanted into a sternocleidomastoid muscle pocket according to standard surgical practice. A histological confirmation of the transplanted parathyroid gland was not performed.

Hence, the first outcome of this study was evaluating the association between the number of intraoperatively identified parathyroid glands and the onset of postoperative hypocalcemia following total thyroidectomy. The secondary outcomes included the impact of accidental parathyroid gland removal confirmed by histology and the effect of parathyroid autotransplantation on postoperative hypocalcemia.

Continuous variables were expressed as means (± standard deviations) or medians (interquartile ranges [IQR]) for non-normal distributions. Categorical variables were summarized as percentages. Chi-squared test and Fisher’s exact test assessed associations between categorical variables, while the Wilcoxon rank-sum test analyzed non-normally distributed continuous variables. Binomial and multinomial logistic regression models were employed to assess the impact of intraoperative parathyroid gland identification and histological findings on the likelihood of developing hypocalcemia. Logistic regression models were also applied to explore predictors of complications, reporting odds ratios (OR) with 95% confidence intervals (CI). All statistical analyses were conducted using R (version 4.1) and Jamovi statistical software (version 2.3). A significance threshold of *p* < 0.05 was used for all analyses. This study was conducted in compliance with institutional and federal ethical guidelines (EKZN Number: 2023 − 00682).

## Results

Overall, 615 patients who underwent thyroid surgery between 2007 and 2022 were included in this study. Of these, 322 patients (52.3%) underwent total thyroidectomy and were included in the analysis. The mean age of the cohort was 52.8 years (± 15.2), with a predominance of female patients (*n* = 213, 66.1%). Most patients presented with benign thyroid diseases, including multinodular goiter (*n* = 161, 50.0%) and uninodular goiter (*n* = 21, 6.5%). Graves’ disease accounted for 22.4% (*n* = 72) of cases. Thyroid malignancies were preoperatively suspected in 14.9% (*n* = 48) of the cohort, with the most frequent subtype being papillary carcinoma (*n* = 34, 10.6%), followed by follicular carcinoma (*n* = 7, 2.2%), medullary carcinoma (*n* = 5, 1.6%), and anaplastic carcinoma (*n* = 2, 0.6%). Baseline characteristics are summarized in Table 1. The recurrent laryngeal nerve was identified in 89.1% (*n* = 287) of cases, while the vagus nerve was identified in a smaller proportion of patients (29.5%, *n* = 95). Intraoperative nerve monitoring (IONM) was used in 82.6% (*n* = 266) of the procedures. Postoperative complications within the first month were detected in 42.9% (*n* = 138) of cases, whereas only 2.8% (*n* = 9) of patients presented a complication more than 30 days after the procedure. Temporary recurrent laryngeal nerve lesions were reported in 5.6% (*n* = 18) of patients, while persistent nerve lesions were observed in 1.6% (*n* = 5). Revision surgeries for postoperative bleeding were also reported in 1.9% (*n* = 6) of cases (Table 2).

**Table 1:** Baseline demographic and clinical characteristics

Characteristic	Total (*N* = 322)
Age, years, mean (SD)	52.8 (15.2)
Sex, n (%)	
Female	213 (66.1)
Previous thyroid surgery, n (%)	12 (3.7)
Preoperative diagnosis, n (%)	
Multinodular goiter	161 (50.0)
Uninodular goiter	21 (6.5)
Graves’ disease	72 (22.4)
Thyroid malignancy (suspected)	48 (14.9)
Papillary carcinoma	34 (10.6)
Follicular carcinoma	7 (2.2)
Medullary carcinoma	5 (1.6)
Anaplastic carcinoma	2 (0.6)
Comorbidities, n (%)	
Hypertension	76 (23.6)
Obesity (BMI ≥ 30)	32 (9.9)
Hyperlipidemia	28 (8.7)

**Table 2:** Intraoperative findings and postoperative outcomes

Variable	Value
Nerve identification, n (%)	
Recurrent laryngeal nerve	287 (89.1)
Vagus nerve	95 (29.5)
Use of Intraoperative nerve monitoring, n (%)	266 (82.6)
Postoperative complications, n (%)	
Within 30 days	138 (42.9)
After 30 days	9 (2.8)
Recurrent laryngeal nerve injury, n (%)	
Temporary	18 (5.6)
Persistent	5 (1.6)
Revision surgery for bleeding, n (%)	6 (1.9)

Intraoperative identification of parathyroid glands was described only in 288 out of the 322 total thyroidectomies (89.4%). In the remaining 34 cases, the operative reports did not explicitly document the number of parathyroid glands identified intraoperatively. One gland was intraoperatively identified in 11.5% (*n* = 33) of the cases, two in 25% (*n* = 72) and three in 22.2% (*n* = 64). The identification of all four glands occurred in 32.3% (*n* = 93) of patients. Among patients with intraoperative identification (*n* = 288), 124 patients (43%) experienced temporary hypocalcemia while 3 (0.9%) experienced persistent hypocalcemia. Among the 3 patients (0.9%) with persistent hypocalcemia, the mean number of parathyroid glands identified intraoperatively was 3.00 (± 1.00). The average number of parathyroid glands identified intraoperatively did not differ significantly between the patients with postoperative hypocalcemia compared to the patients with normal levels of calcium (*p* = 0.594, Table 3). No significant association was observed between accidental removal and the occurrence of postoperative hypocalcemia (*p* = 0.402).

**Table 3:** Comparison of parathyroid gland identification and accidental removal in patients with and without postoperative hypocalcemia

Variable	Hypocalcemia (*n* = 124)	No hypocalcemia (*n* = 164)	*p*-value
Mean number of identified parathyroid glands	2.54 ± 1.27	2.60 ± 1.31	0.594
Accidental gland removal (%)	47.3%	40.5%	0.402

Among the 322 total thyroidectomies, no parathyroid glands were detected in the histological specimens in 81.4% (*n* = 262) of cases, while one gland was described in 17.1% of cases (*n* = 55) and two in 1.6% of cases (*n* = 5). There was no significant association between intraoperative identification and histological identification of parathyroid glands in the pathological analysis (*p* = 0.177). 106 patients (40.5%) with no parathyroid glands identified in the histological analysis developed temporary hypocalcemia. In 26 (20.4%) of the patients where parathyroid glands were identified during surgery and that postoperatively experienced hypocalcemia, one parathyroid gland was found in the histological specimen, while in another patient (0.9%) two glands were detected.

Autotransplantation was performed on 41 (12.7%) patients during total thyroidectomy. Complete data on autotransplantation and postoperative hypocalcemia was available only for 305 patients (94.7%). Comparing the onset of postoperative hypocalcemia in patients who underwent parathyroid autotransplantation or not, the analysis showed that autotransplantation had a significantly higher rate of postoperative hypocalcemia compared with those without autotransplantation (61.0% vs. 38.6%; OR 2.48, 95% CI 1.26–4.87; *p* = 0.007, Table 4). In multivariable logistic regression analysis, parathyroid autotransplantation remained independently associated with temporary hypocalcemia (adjusted OR 2.45, 95% CI 1.20–5.00; *p* = 0.015).

**Table 4:** Association between parathyroid autotransplantation and postoperative hypocalcemia in patients with available data (*n* = 305)

	No Hypocalcemia (*n* = 178)	Hypocalcemia (*n* = 127)	OR	95% CI	*p*-value
Parathyroid autotransplantation					
No	162 (61.4%)	102 (38.6%)	Reference		
Yes	16 (39.0%)	25 (61.0%)	2.48	1.26 − 4.87	0.007

Finally, to identify factors associated with postoperative hypocalcemia, univariate and multivariable logistic regression analyses were performed (Tables 5 and 6). In univariate analysis, none of the evaluated variables, including the number of intraoperatively identified parathyroid glands, showed a significant association with temporary hypocalcemia. In multivariable logistic regression analysis including age, sex, number of identified parathyroid glands, and parathyroid autotransplantation, only autotransplantation was independently associated with an increased risk of temporary hypocalcemia (OR 2.45, 95% CI 1.19–5.05, *p* = 0.015). No significant association was observed for the number of identified glands.

**Table 5:** Univariate logistic regression for temporary hypocalcemia after total thyroidectomy

Identified Parathyroid glands	OR	95% CI	*p*-value
1 vs. 0	1.01	0.36–2.84	0.993
2 vs. 0	0.92	0.37–2.28	0.857
3 vs. 0	1.45	0.58–3.64	0.427
4 vs. 0	0.79	0.32–1.90	0.594

**Table 6:** Multivariable logistic regression for temporary hypocalcemia after total thyroidectomy

Variable	OR	95% CI	*p*-value
Age (per year)	1.01	0.99–1.02	0.12
Female sex	1.18	0.72–1.93	0.51
Number of parathyroid glands identified (per gland)	0.97	0.80–1.18	0.78
Parathyroid autotransplantation	2.45	1.19–5.05	0.015

## Discussion

This study provides an in-depth evaluation of postoperative hypocalcemia following thyroid surgery, focusing on the influence of parathyroid gland identification and preservation. Postoperative hypocalcemia remains a significant concern following thyroidectomy, with our study showing a temporary hypocalcemia rate of 43% and a low incidence of persistent hypocalcemia (0.9%). These findings align with previous literature, where transient hypocalcemia affects between 20% and 50% of patients, while persistent hypocalcemia is observed in 1–4% of cases [14, 15]. The identification and preservation of parathyroid glands during surgery plays a crucial role in mitigating this complication.

Our data demonstrated that intraoperative identification of parathyroid glands was achieved in 89.4% of total thyroidectomies, yet no significant association was found between intraoperative gland identification and the risk of postoperative hypocalcemia (*p* = 0.594). Accordingly, prior studies suggested that visual identification alone may not be sufficient to prevent hypocalcemia, as factors such as gland viability and vascularization also influence postoperative calcium homeostasis [16]. The role of anatomical landmarks in parathyroid gland preservation has been previously emphasized, indicating that careful surgical dissection and experience significantly impact outcomes [17].

The study also examined the incidence of accidental parathyroid gland removal, which was histologically identified in 18.6% of cases. Although there was a trend toward a higher risk of temporary hypocalcemia in patients with histologically confirmed inadvertent gland excision (47.3% vs. 40.5%), the association was not statistically significant (*p* = 0.402). These results support findings from previous reports indicating that unintentional gland removal contributes to hypocalcemia risk but may not be the sole determinant [18]. Moreover, no significant correlation was found between intraoperative identification of glands and their accidental removal, reinforcing the idea that intraoperative visual identification alone does not guarantee preservation.

The relationship between parathyroid autotransplantation and hypocalcemia was also investigated. Among patients undergoing total thyroidectomy, parathyroid autotransplantation was performed in 41 patients, of whom 61.0% developed postoperative hypocalcemia compared to 38.6% of patients without autotransplantation (*p* = 0.007), indicating a significant association with transient hypocalcemia likely related to the period required for graft revascularization and functional recovery. Other authors similarly indicated that autotransplantation, while a valuable technique for preserving parathyroid function, may initially contribute to transient hypocalcemia due to the time required for graft revascularization and functionality [19]. Wang et al. observed that transplanted glands often require weeks to regain full functionality, which can lead to transient hypocalcemia during the recovery period [20]. In essence, autotransplantation should be reserved for cases where parathyroid gland viability is clearly compromised, rather than as a routine precautionary measure. A study by Palazzo et al. also found that the incidence of temporary hypocalcemia increased with the number of glands transplanted [21], supporting the idea that autotransplantation should be done only when necessary and with as few glands as possible. Notably, patients with Graves’ disease and multinodular goiter are more likely to require autotransplantation, possibly due to the surgical difficulty in preserving parathyroid glands in these cases. Literature suggests that inflammatory and hypervascularized thyroid diseases increase the risk of parathyroid gland devascularization [22]. Therefore, the link between parathyroid autotransplantation and transient hypocalcemia should be viewed with caution, as it may indicate surgical complexity rather than a direct cause-and-effect relationship.

Comparing our findings with prior research, we observed a relatively low rate of persistent hypocalcemia (0.9%). It is important to note that some patients may maintain normocalcemic levels through calcium or vitamin D supplementation. Therefore, the reported rate likely reflects biochemical persistent hypocalcemia rather than the full spectrum of clinically treated postoperative hypoparathyroidism. A low rate of persistent hypocalcemia could however be attributed to meticulous surgical techniques, the use of intraoperative nerve monitoring in 82.6% of cases, and careful parathyroid gland handling. This is in line with studies highlighting the role of surgical expertise and perioperative management in reducing long-term complications [23].

At the time of our study no advanced intraoperative tools were available in our center to perform intraoperative parathyroid gland identification. It is essential to consider how our results might have differed if new surgical techniques like NIRAF had been used. A multicenter randomized clinical trial found that the use of NIRAF during total thyroidectomy significantly lowered the rate of temporary postoperative hypocalcemia from 22% to 9% [24]. However, a randomized controlled trial in Germany found that it did not significantly reduce postoperative hypoparathyroidism [25]. While NIRAF shows great promise, it is not yet universally available, and its effectiveness in preventing hypoparathyroidism remains a subject for further investigation.

While our study provides valuable insights, it is limited by its retrospective design, which introduces potential selection and reporting biases. Another limitation is the incomplete documentation of intraoperative parathyroid identification in a small subset of total thyroidectomy cases (*n* = 34), which may reflect variability in operative reporting over the long study period. During this period, thyroidectomies were performed by different experienced surgeons within the same institution following standardized surgical principles, although minor variations in individual surgical technique over time cannot be completely excluded. Also, variables such as underlying thyroid pathology, extent of surgery (e.g., central neck dissection), reoperative procedures, and overall surgical complexity may influence postoperative calcium homeostasis and were not fully controlled for in our analysis. However, its strengths include a large sample size for Switzerland (615 patients) and a long follow-up period (15 years), enhancing the reliability of our findings.

In conclusion, it is essential to maintain a high level of surgical skill in identifying and preserving as many parathyroid glands as possible through traditional methods, ensuring the best possible surgical outcomes even in the absence of advanced intraoperative tools. Meticulous surgical techniques and personal surgical experience remain essential for minimizing postoperative hypocalcemia. While intraoperative parathyroid gland identification and autotransplantation play crucial roles, their impact on hypocalcemia risk is multifactorial. Further research is warranted to refine surgical protocols and integrate emerging technologies to improve patient outcomes. Also, how potential confounders such as underlying thyroid pathology, extent of surgery, and surgical complexity may affect postoperative calcium homeostasis should be considered in future prospective studies.

## Data Availability

The datasets used and/or analysed during the current study are available from the corresponding author on reasonable request.
